# Expansion of Liver Transplantation Criteria for Hepatocellular Carcinoma from Milan to UCSF in Australia and New Zealand and Justification for Metroticket 2.0

**DOI:** 10.3390/cancers14112777

**Published:** 2022-06-03

**Authors:** Savio G. Barreto, Simone I. Strasser, Geoffrey W. McCaughan, Michael A. Fink, Robert Jones, John McCall, Stephen Munn, Graeme A. Macdonald, Peter Hodgkinson, Gary P. Jeffrey, Bryon Jaques, Michael Crawford, Mark E. Brooke-Smith, John W. Chen

**Affiliations:** 1 South Australia Liver Transplant Unit, Flinders Medical Centre, Adelaide, SA 5042, Australia; savio.barreto@flinders.edu.au (S.G.B.); mark.brooke-smith@sa.gov.au (M.E.B.-S.); 2 Australian National Liver Transplant Unit, Royal Prince Alfred Hospital, University of Sydney, Sydney, NSW 2050, Australia; simone.strasser@health.nsw.gov.au (S.I.S.); g.mccaughan@centenary.org.au (G.W.M.); michael.crawford1@health.nsw.gov.au (M.C.); 3 Austin Health, Heidelberg, VIC 3081, Australia; mafink@unimelb.edu.au (M.A.F.); robert.jones@austin.org.au (R.J.); 4 Department of Surgery, The University of Melbourne, Melbourne, VIC 3010, Australia; 5 Auckland City Hospital, Auckland 1023, New Zealand; johnmc@adhb.govt.nz (J.M.); professormunn@gmail.com (S.M.); 6 Queensland Liver Transplant Service, Princess Alexandra Hospital, Woolloongabba, QLD 4102, Australia; g.macdonald@uq.edu.au (G.A.M.); peter.hodgkinson@health.qld.gov.au (P.H.); 7 Sir Charles Gairdner Hospital, Nedlands, WA 6009, Australia; gary.jeffrey@uwa.edu.au (G.P.J.); bryon.jaques@health.wa.gov.au (B.J.)

**Keywords:** hepatitis, outcomes, survival, Metroticket 2.0, Milan, UCSF

## Abstract

**Simple Summary:**

Liver transplantation (LT) is considered the only curative therapeutic option for early, unresectable, and unablatable hepatocellular carcinoma (HCC), particularly in the setting of chronic liver disease. The criteria for selecting patients for LT for HCC have evolved since the description of the Milan Criteria by Professor Mazzaferro. In Australia and New Zealand (ANZ), the choice of criteria has expanded over the last 24 years from the Milan to the University of California San Francisco (UCSF) criteria and, more recently, to Metroticket 2.0 (MT2). This study analysed the overall and HCC-related deaths following LT in ANZ through the last 24 years to clarify the impact of the expansion of these criteria. Our data confirm that overall survival following LT for HCC has significantly improved over time despite expanding criteria from Milan to UCSF. Patients fulfilling the MT2 criteria have a survival comparable to the UCSF cohort. Thus, the expansion of criteria to MT2 is justifiable.

**Abstract:**

Background: Expansion in liver transplantation (LT) criteria for HCC from Milan to UCSF has not adversely impacted overall survival, prompting further expansion towards Metroticket 2.0 (MT2). In this study, we compared patient survival post-transplant before and after 2007 and long-term outcomes for LT within Milan versus UCSF criteria (to determine the true benefit of the expansion of criteria) and retrospectively validated the MT2 criteria. Methods: Retrospective analysis of ANZLITR (including all patients transplanted for HCC since July 1997). The entire cohort was divided based on criteria used at the time of listing, namely, *Milan era* (1997–2006) and the *UCSF* *era* (2007–July 2015). Results: The overall 5- and 10-year cumulative survival rates for the entire cohort of 691 patients were 78% and 69%, respectively. Patients transplanted in *UCSF era* had significantly higher 5- and 10-year survival rates than in the *Milan era* (80% vs. 73% and 72% vs. 65%, respectively; *p* = 0.016). In the *UCSF era*, the 5-year survival rate for patients transplanted within Milan criteria was significantly better than those transplanted outside Milan but within UCSF criteria (83% vs. 73%; *p* < 0.024). Patients transplanted within the MT2 criteria had a significantly better 5- and 10-year survival rate as compared to those outside the criteria (81% vs. 64% and 73% vs. 50%, respectively; *p* = 0.001). Conclusion: Overall survival following LT for HCC has significantly improved over time despite expanding criteria from Milan to UCSF. Patients fulfilling the MT2 criteria have a survival comparable to the UCSF cohort. Thus, expansion of criteria to MT2 is justifiable.

## 1. Introduction

The incidence of hepatocellular cancer is increasing worldwide [1,2]. It has been predicted that these trends are likely to persist over the next couple of decades [1]. Liver transplantation (LT) is considered the only curative therapeutic option for early, unresectable, and unablatable hepatocellular carcinoma (HCC), particularly in the setting of chronic liver disease [3].

Following the proposal of the Milan [4] criteria for listing patients with HCC for LT, it was noted that approximately 25% of patients were found to be outside the criteria post-LT on the explant histology, despite which their 5-year survival was above 50% [5]. This prompted the initial expansion of the criteria for LT for HCC from the more conservative Milan [4] criteria to the University of California San Francisco (UCSF) [6] criteria. In doing so, the perceived improvement in survival of HCC with LT was sustained despite this moderate expansion of the criteria [6]. Thereafter, a number of countries proposed criteria beyond the UCSF to list patients for LT, especially those patients receiving grafts from living donors (LDLT) [7,8,9,10].

In 2007, we altogether moved to adopt the UCSF criteria (based on radiological findings) for the listing of patients for LT for HCC in Australia and New Zealand (ANZ). This was based on our initial results on the safety of applying the UCSF criteria [11]. A more pressing question that remained unanswered was whether the expansion in the criteria adversely affected the overall outcomes of LT for HCC. From the start of 2020, the Metroticket 2.0/MT2 [12] has been used as the criteria to list patients for LT within ANZ, considering that they have been shown to outperform the Milan, UCSF, up-to-seven criteria (*p* < 0.001), and AFP French model (*p* = 0.044) to predict which patients will survive for five years after LT [13,14]. MT2 does not define strict/definite criteria for patient inclusion but provides a model to predict tumour-related death. It is thus a prognostic index developed under a competing-risk framework and allows to estimate the HCC-specific survival starting from radiological staging and serum α-fetoprotein (α-FP) value [13].

The aims of the present study were as follows:

(a) to compare the long-term outcomes of patients with HCC transplanted before 2007 and after 2007, when the UCSF was the sole criterion used for listing patients for LT for HCC;
(b) to compare the long-term outcomes for LT within the Milan versus the UCSF criteria in order to determine the true benefit of the expansion of the criteria;
(c) to retrospectively validate the MT2 criteria [12]. This was done to explore the possibility for further expansion of the criteria for LT for HCC in ANZ.

## 2. Materials and Methods

A retrospective analysis of the ANZ Liver and Intestinal Transplant Registry was performed on all patients who were listed for LT for HCC in ANZ from July 1997 until July 2015. The American Association for the Study of Liver Diseases criteria were used to diagnose patients with HCC radiologically. Nearly all transplants were performed using deceased organs. Data examined included details of patient demographics, disease etiology, as well as post-operative outcomes, including histopathological findings, overall patient survival, the cumulative incidence of HCC-related deaths, and cause of death. This study, using data derived from the ANZ Liver and Intestinal Transplant Registry, abides by the Ethical Principles for Medical Research Involving Human Subjects outlined in the 2013 Declaration of Helsinki.

Pre-transplant variables (at the time of listing) analysed included age, sex, serum α-FP levels, and radiological tumour size and number at the time of listing. Pre-LT treatment of the tumour by any modality was noted but not analysed, since details of these treatments were not elaborated on in the Registry dataset. These factors were analysed with respect to patient overall survival. The Milan and UCSF Criteria, as previously published [4,6], were applied to this cohort of patients using both pre-LT (radiological) and post-LT (pathological) classification.

The effect of the era of LT on patient survival was evaluated. The entire cohort was divided into two eras based on the criteria adopted by the Transplantation Society of ANZ (TSANZ) at the time of listing, namely, the *Milan era* (1997–2006) and the *UCSF era* (2007 to July 2015). The criteria for listing patients for LT in ANZ are strict and based on pre-transplant radiology. However, it is possible that disease progression while on a waiting list and (radiological) under-staging may contribute to patients being noted to lie outside criteria in explant pathology.

### 2.1. Metroticket 2.0 Framework

The sum of number of tumours and diameter of the largest tumour (in centimetres) was considered as one tumour parameter (i.e., 1 nodule plus largest tumour size 5 cm = 6). The α-FP value before LT, submitted to the Registry, was considered the second parameter in the MT2 criteria. The 2 parameters were combined into 3 groups:

Group 1: Sum of the number and size of tumours <7 and α-FP <200 ng/mL;

Group 2: Sum of the number and size of tumours <6 and α-FP 200–400 ng/mL;

Group 3: Sum of the number and size of tumours <5 and α-FP 400–1000 ng/mL;

Patients who fell into either MT2 groups 1–3 were predicted to have a 70% chance of HCC-specific survival 5 years after LT.

Patients were considered outside the MT2 criteria (Group 4) if: (a) The sum of number and size of the largest tumour was ≥7 or (b) α-FP > 1000 mg/mL, or (c) α-FP 200–400 ng/mL (group 2) but with the sum of number and size of the largest tumour ≥ 6.0, or (d) α-FP 400–1000 ng/mL (Group 3) but with the sum of number and size of the largest tumour ≥ 5.0 [13].

The analysis of the MT2 criteria was performed using post-LT (pathological) information on all patients in whom the required information was available.

### 2.2. Statistical Analysis

Survival was calculated based on the last date of follow-up as of 31 December 2019. Statistical analysis was performed using IBM^®^ SPSS^®^ Statistics for Windows, version 25 (IBM Statistical Package for the Social Sciences-SPSS; IBM, Corp Armonk, NY, USA). Kaplan–Meier estimates were used to analyse the survival following LT for HCC in patients within the ANZ Liver and Intestinal Transplant Registry since 1997. The data are expressed as mean (±standard deviation (SD)) and/or median (interquartile range (IQR)). The Mann–Whitney U-test and the Pearson Chi-square test were used to compare continuous and categorical variables. The log-rank test compared survival within and outside of the liver transplant criteria: Milan, UCSF, or MT2; and survival between patient demographics and clinical features. Further investigation with Cox proportional hazards regression analysis was used to study the effects of age, gender, α-FP, underlying disorders, and hepatitis status on survival and the risk of death. Competing risk analysis was performed, examining HCC-related death as the event of interest. The non-HCC related deaths were considered competing events. Patients were censored at date of loss to follow-up or end of follow-up. Fine and Gray [15] sub-distribution hazard models were used via cumulative incidence function and sub-distribution hazard functions to assess the sub-distribution hazard ratios (SHR) of cause-specific mortality. The cumulative incidence curves were plotted from the sub-distribution hazard model. A *p*-value <0.05 was considered statistically significant.

## 3. Results

Since July 1997, a total of 691 patients underwent LT for HCC (including those who were transplanted for decompensated cirrhosis who also had HCC) within the 6 LT units in Australia and New Zealand. This represented 21% of all adult liver transplants (*n* = 3222) in this period. Of this cohort, 137 patients had been included in a previous publication [11].

### 3.1. Patient Characteristics 

The overall cohort (Table 1) included a majority of male patients (86%) with a median patient age of 55.7 years (IQR 51.5–60.1 years), 53% being hepatitis C antibody positive, with a median serum α-FP level of 12 ng/mL (IQR 5.0–44.0 ng/mL).

In the *Milan era* (*n* = 254), there was a male predominance (86%) with a median patient age of 54.7 years (IQR 49.1–58.8 years), 44% hepatitis C antibody positivity, and a median serum α-FP level of 17 ng/mL (IQR 6.0–80.7 ng/mL).

In the *UCSF era* (*n* = 437), the male predominance (86%) persisted with a median patient age of 56.4 years (IQR 52.2–60.4 years), 57% hepatitis C positivity, and a median α-FP level of 9.8 ng/mL (IQR 4.0–28.4 ng/mL).

### 3.2. Survival in the Milan and UCSF Eras

**Figure 1:**
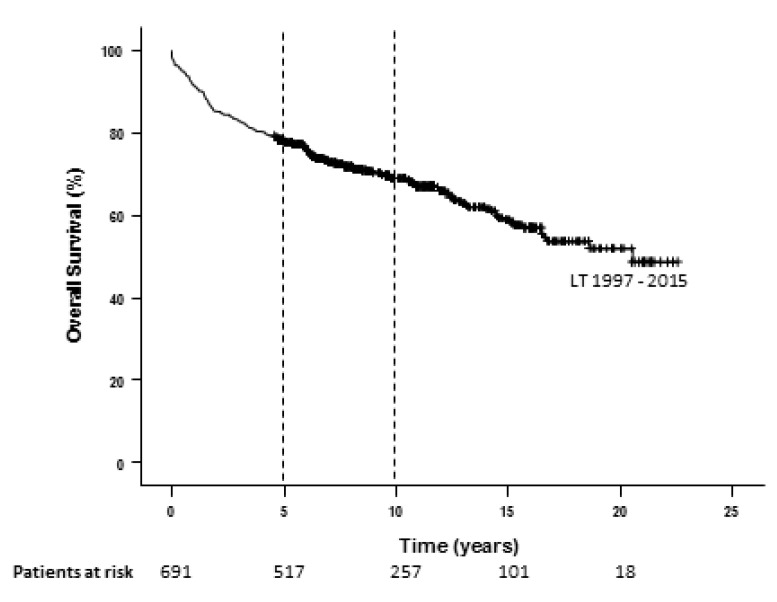
Kaplan-Meier curve for overall survival for HCC following LT in Australia and New Zealand since 1997.

The overall 5- and 10-year Kaplan–Meier survival rates for the entire cohort of patients were 78% and 69%, respectively (Figure 1). There were 49% of patients alive at 20 years.

Patients undergoing LT who were <55 years of age, had significantly better Kaplan– Meier5- and 10-year survival rates compared to those ≥55 years of age (81% vs. 75% and 72% vs. 67%, respectively; *p* = 0.017). Patients with underlying alcohol-related cirrhosis had a significantly worse survival rate compared to those with viral hepatitis at 5- and 10-years (78% vs. 72% and 71% vs. 57%, respectively; *p* = 0.014). However, amongst those patients with viral hepatitis, patients with hepatitis C had significantly worse survival rates than those with hepatitis B infection at 5- and 10-years (82% vs. 76% and 79% vs. 67%, respectively; *p* = 0.007). This is likely the result of all the patients being transplanted prior to the introduction of direct-acting anti-viral (DAA) therapy. Other factors noted to be associated with significantly better 5- and 10-year survival were serum α-FP levels <10 ng/mL compared to ≥10 ng/mL [16] (79% vs. 75% and 73 vs. 63%, respectively; *p* = 0.011); as well as capsular (79% vs. 61% and 70% vs. 48%, respectively; *p* = 0.0001) and (macro- and micro-) vascular (80% vs. 59% and 72% vs. 46%, respectively; *p* = 0.0001) invasion (Table 2).

Gender, lymph node metastases, and recipient blood group did not influence survival rates. Cox Regression stepwise analysis showed that advancing age at LT is a predictor of reduced survival where yearly increase in age increases the hazard rate by 2.7% (95% CI hazard ratio = 1.003 to 1.052; *p* = 0.027) (Table 3).

**Figure 2:**
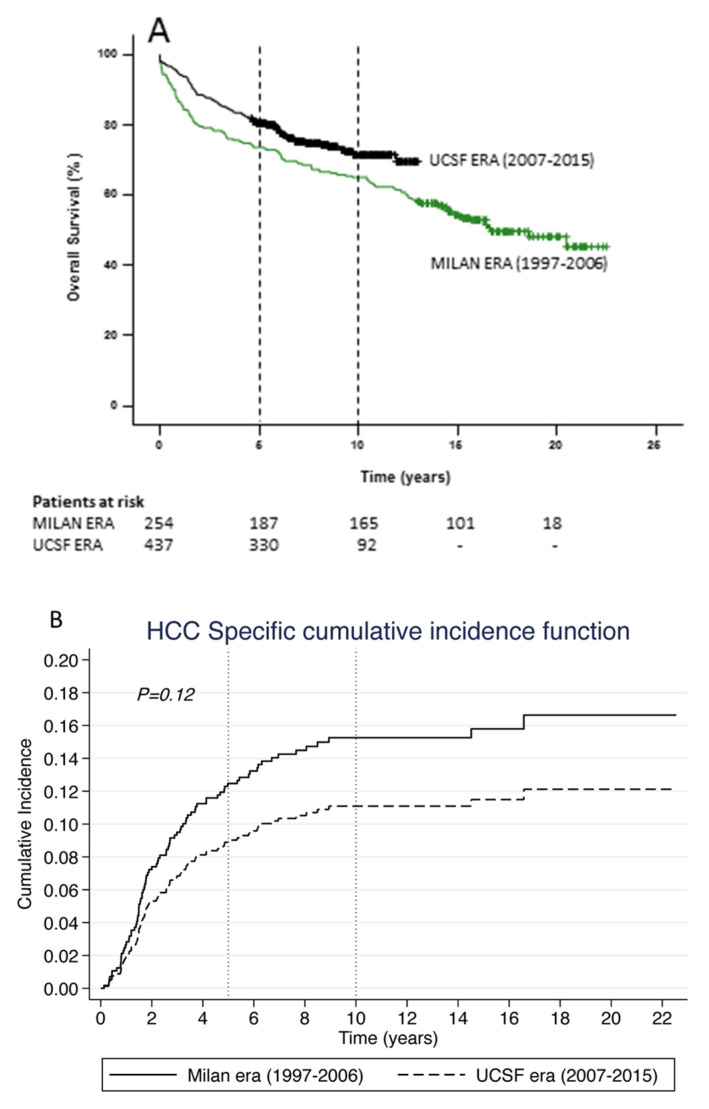
Kaplan– Meier Curves for (**A**) Overall survival for HCC following LT comparing the *Milan era* (green line) vs. the *UCSF era* (black line), and (**B**) HCC-related death following LT comparing the *Milan era* (solid line) vs. the *UCSF era* (dashed line).

Patients transplanted in the *UCSF era* had significantly higher 5- and 10-year overall survival rates (80% vs. 73% and 72% vs. 65%, respectively; *p* = 0.016) (Figure 2A). Although not significant, cumulative incidence of HCC-related death was 29% lower (SHR = 0.71, 95% CI 0.46–1.09, *p* = 0.12) in the *UCSF* era (2007–2015) compared to the *Milan* era (1997–2006). The 5- and 10-year cumulative incidence of deaths were almost similar to overall incidence of death (Figure 2B).

### 3.3. Was the Survival Advantage in the UCSF Era due to Patients Transplanted within the Milan Criteria?

**Figure 3:**
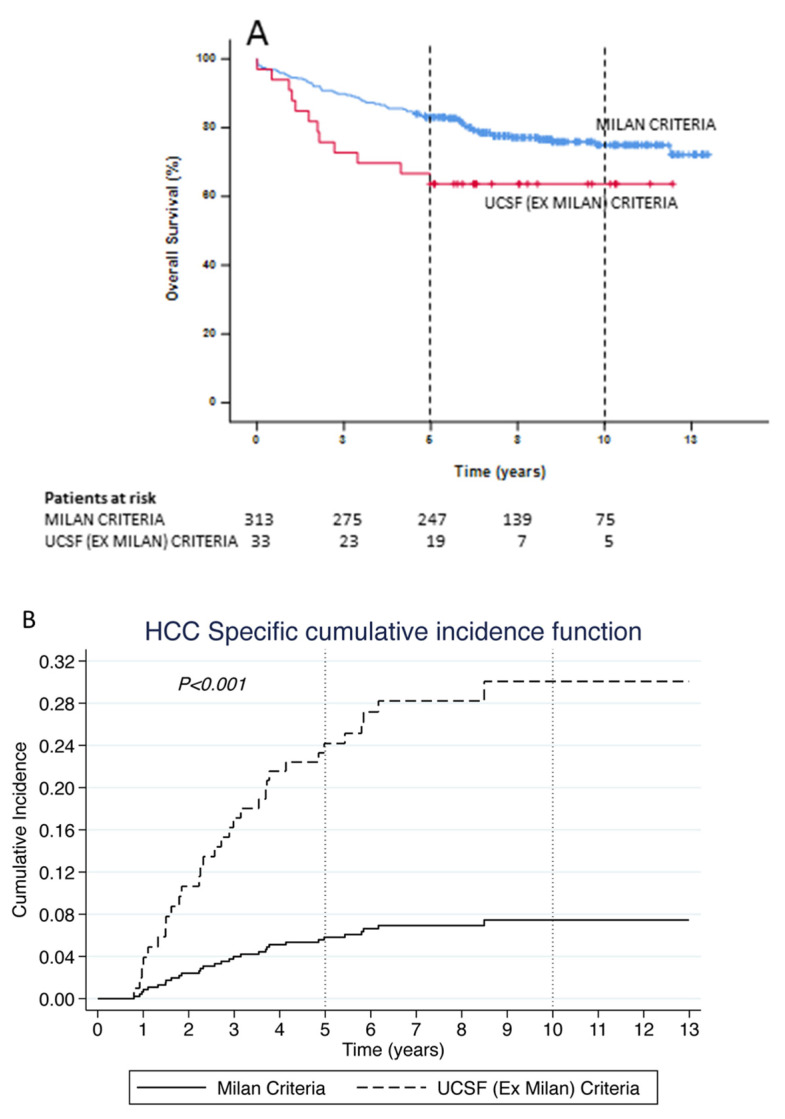
Kaplan–Meier Curves for (**A**) Overall survival for HCC following LT in the *UCSF era* comparing those transplanted within the Milan criteria (blue line) vs. those transplanted within UCSF but outside the Milan criteria (red line) and (**B**) HCC-related death following LT in the *UCSF era* comparing those transplanted within the Milan criteria (solid line) versus those transplanted within UCSF but outside the Milan criteria (dashed line).

In the *UCSF era*, the survival rates of patients transplanted within the Milan criteria (*n* = 313) were significantly better than the survival rates of those patients transplanted outside Milan but within UCSF criteria (*n* = 124) at 5- and 10-years (83% vs. 73% and 75% vs. 62%, respectively; *p* < 0.024) (Figure 3A). The cumulative incidence of HCC-related death was 4.6 times higher (SHR = 4.61, 95% CI 2.11–10.11, *p* < 0.001) for patients transplanted in the *UCSF era* who were outside Milan (Ex Milan) criteria but with UCSF criteria compared to those within Milan criteria. The 5-year cumulative incidence of death was almost 6 times higher (SHR = 5.75, 95% CI 2.57–12.90, *p* < 0.001) for those transplanted with UCSF (Ex Milan) criteria compared to those who were within Milan criteria. However, the 10-year cumulative incidence of deaths was almost similar to the overall incidence of death (Figure 3B).

### 3.4. Validating the MT2 Criteria

**Figure 4:**
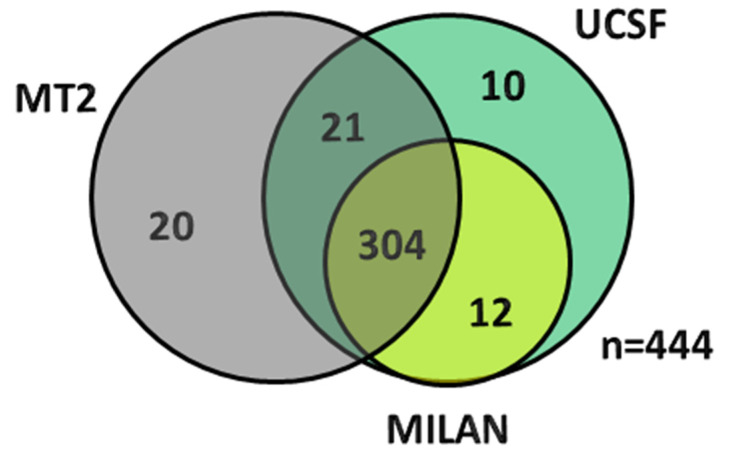
Venn diagram depicting the overlap of patients across the different criteria (Milan, UCSF and Metroticket 2.0) based on the re-categorisation of patients based on the Metroticket 2.0 using post-LT (pathological) information on all patients (77 patients were found to lie outside all the criteria).

When validating the MT2 criteria using the patients in the Registry, 345 patients were considered ‘within’ the prescribed criteria, while 99 patients were outside the criteria because of the serum α-FP level being >1000 ng/mL (*n* = 15); sum of tumour number and maximum tumour size >7 (*n* = 81); serum α-FP level between 200–400 ng/mL and sum of number and maximum tumour size > 6 (*n* = 1); or serum α-FP level between 400–1000 ng/mL and sum of number and maximum tumour size >5 (*n* = 2) (Figure 4).

**Figure 5:**
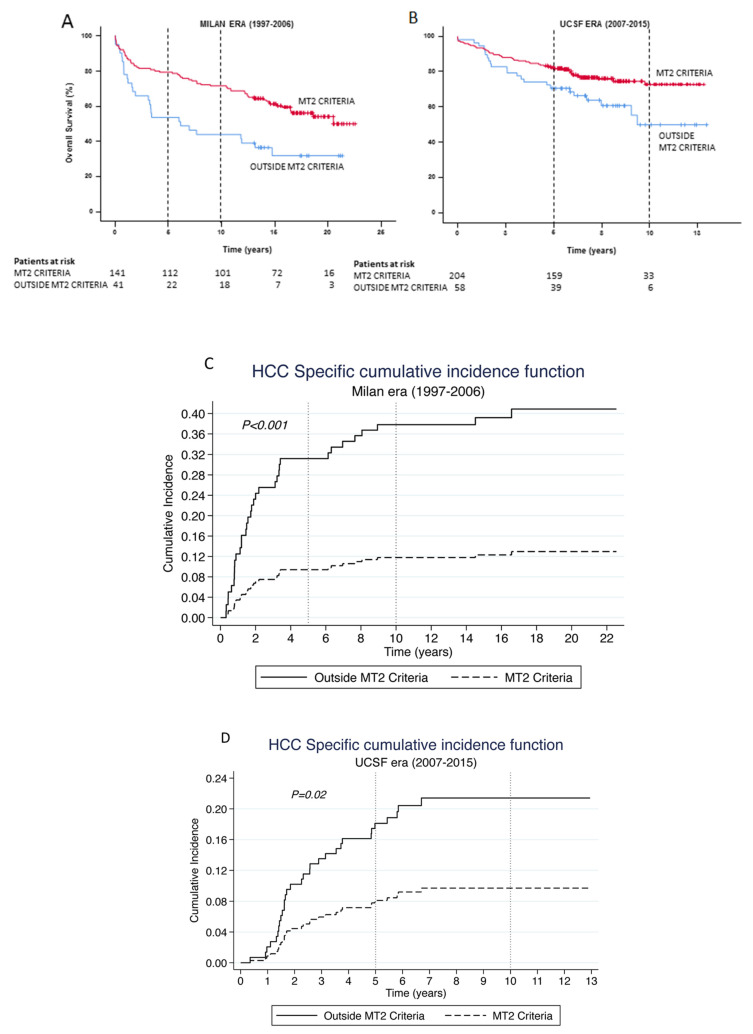
Kaplan–Meier Curves for Overall survival for HCC following LT comparing those within the Metroticket 2.0 criteria (red line) vs. those outside the criteria (blue line) within the (**A**) *Milan* and (**B***) UCSF eras*, and HCC-related death following LT comparing those within the Metroticket 2.0 criteria (bold line) vs. those outside the criteria (dashed line) within the (**C**) *Milan* and (**D**) *UCSF eras*.

Patients transplanted within the MT2 criteria had a significantly better survival rate at 5- and 10-years as compared to those outside the criteria (81% vs. 64% and 73% vs. 50%, respectively; *p* = 0.001). The survival advantage is maintained across both eras, namely, *Milan era* and *UCSF era*. Figure 5A demonstrates a significant overall survival advantage at 5- and 10-years for those patients transplanted within what is now considered to be the MT2 criteria (*Milan era:* 79% vs. 54% and 72% vs. 44%, *p* = 0.001, respectively; *UCSF era:* 82% vs. 71% and 73% vs. 50%, *p* = 0.017, respectively). The cumulative incidence of HCC-related death was 74% lower (SHR = 0.26, 95% CI 0.13–0.51, *p* < 0.001) amongst patients transplanted within the MT2 criteria in the *Milan* era compared to those outside MT2 criteria. The 5- and 10-year cumulative incidence of deaths were also significantly lower amongst patients transplanted within the MT2 criteria compared to those outside criteria [SHR = 0.18, 95% CI 0.08–0.40, *p* < 0.001 and SHR = 0.24, 95% CI 0.12–0.48, *p* < 0.001, respectively]. Similarly, the cumulative incidence of HCC-related death was 58% lower (SHR = 0.42, 95% CI 0.21–0.87, *p* < 0.019) amongst patients transplanted within the MT2 criteria in the *UCSF* era compared to those outside MT2 criteria. The 5- and 10-year cumulative incidence of deaths were also significantly lower amongst patients transplanted within the MT2 criteria compared to those outside criteria [SHR = 0.45, 95% CI 0.21–0.99, *p* < 0.048 and SHR = 0.42, 95% CI 0.21–0.87, *p* < 0.019, respectively] (Figure 5B).

## 4. Discussion

These results indicate that overall survival following LT for HCC in the ANZ context has significantly improved over time despite the expansion of the criteria (to UCSF in 2007 [11]). Patients transplanted within the Milan criteria have significantly better survival than those transplanted outside the Milan criteria but within the UCSF criteria. The MT2 criteria were validated, but the outcomes are comparable to those of UCSF.

Patient survival has certainly improved over time following LT [17]. This improvement has been attributed to various factors, including better patient selection with optimisation of transplant recipients and donors, delisting patients who progress to outside criteria, improvements in surgical technique and post-operative care, advances in organ procurement and preservation, better immunosuppression (including the use of mTOR inhibitors), and improved prevention and management of recurrent disease (prophylaxis and treatment of HBV and HCV), complications and co-morbidities. Overall survival following LT for HCC, too, has been reported to improve over time [18], and this was attributed to improved perioperative care, increased use of locoregional therapies as a bridge to LT [19], and better patient selection. However, data from the ANZ Liver and Intestinal Transplant Registry indicates that despite expanding the criteria for transplantation, survival continued to improve, supporting a role for general improvements in perioperative care and surgical technique (as outlined above). The lack of a difference in cumulative incidence of HCC-related death between the two eras (Figure 2B) despite a significant difference in overall survival attests to the competing factors (including a significant difference in HCV infections, the advent of DAA, etc.) impacting on the perceived overall survival advantage.

The reduced survival in recipients over 55 years is intriguing. Stratifying age by decade resulted in significantly lower numbers of patients in some decades. Hence, the 55-year cut-off, with 312 patients below the cut-off, and 379 greater than and equal to the cut-off, served as a balanced comparator. Jain et al. noted an overall poor survival amongst LT recipients over 60 years of age (5-year survival rates of 61% versus 67% for those aged 19–60 years) [17]. A similar poor survival in patients with HCC older than 60 years was noted amongst those treated with radiofrequency ablation/RFA (5-year survival rate of 30% vs. 37%, *p* = 0.02) [20]. Whether this is indicative of a poorer tumour biology is uncertain. An analysis of the Surveillance, Epidemiology and End-results (SEER) database indicated that patients older than 60 years tend to have a significantly higher number of tumours >5 cm [21]. Considering that the median age for HCC in Australia is 67.2 years [22] and 81% of patients with HCC in New Zealand are above the age of 50 years [23], this aspect merits further research. One potential explanation for the poorer survival in older patients could be their increased likelihood of co-morbidities. Koczwara et al. [24] have recently demonstrated the association between co-morbidities and survival in cancer.

Our data indicate that the overall survival for patients being transplanted outside Milan but within UCSF was significantly lower than for patients transplanted within Milan criteria (Figure 3). Despite this, the findings from Table 4 [25,26,27,28,29] indicate that the 5-year survival rates in the UCSF era were comparable to the rest of the world. Our study also represents the largest cohort of patients with the longest follow-up (20-year survival rates) following LT for HCC. We were also able to validate the MT2 criteria [12]. Our findings support a robust survival using these criteria when compared to other similar validation studies from around the world [13,30,31].

Expansion of criteria for LT for HCC has not always met with success [32]. However, the findings of our long-term analysis on survival within the ANZ Liver and Intestinal Transplant Registry with 10-year survival data are certainly encouraging and strengthens the move toward expanding criteria in a staged manner with a regular audit of outcomes to ensure that expansion of criteria does not come at the cost of compromised patient survival. We wish to highlight that sentinel work by Centonze et al. [33] and the European Association for the Study of the Liver (EASL) [34] have provided added benefits to the evolution of MT2 with regards to preoperative staging and response to neoadjuvant treatments, respectively.

The ANZ Liver and Intestinal Transplant Registry is able to capture every patient who underwent an LT over the study period, and the information entered is valid. However, being a bi-national registry, the number of data entry parameters is restricted as compared to an individual institution, and thus, detailed analyses of subsets of patients are limited. This is why we do not have detailed pre-LT treatment and donor data that may impact on survival. Another shortcoming of the study is the lack of complete pathologic data from 247 patients (36%), leading to the exclusion of these patients from the overall analysis. However, we do not have reason to believe that this would skew the results in any way. Another limitation of our study is that the analysis is retrospective in nature and based on explant histology to stratify patients within the criteria for the manuscript.

## 5. Conclusions

Overall survival following LT for HCC has significantly improved over time despite expanding the criteria from Milan to UCSF. It is likely that this survival advantage is due to the patients who are listed as UCSF but fall within the Milan criteria. Patients fulfilling the MT2 criteria have a survival comparable to the UCSF group, and further expansion to MT2 appeared justified.

## Figures and Tables

**Table 1:** Patient characteristics (Abbreviation: IQR = interquartile range; SD = standard deviation; NAFLD—non-alcoholic fatty liver disease; α-FP—alpha-fetoprotein).

Demographic Characteristics	Overall Cohort (*n* = 691)	Milan era (*n* = 254) 1997–2006	UCSF era (*n* = 437) 2007–2015	*P* Value (Era Comparison)
Gender male/female *n* (%)	595 (86)/96 (14)	219 (86)/35 (14)	377 (86)/60 (14)	*0.871 ^#^*
Median Age in years (IQR)	55.7 (51.5–60.1)	54.7 (49.1–58.8)	56.4 (52.2–60.4)	0.001 *
Underlying disease *n* (%) Viral hepatitis Alcohol-related disease Immune/Inflammatory /metabolic NAFLD	543 (79) 65 (9) 45 (7) 37 (5)	200 (79) 28 (11) 13 (5) 13 (5)	343 (79) 37 (9) 32 (7) 24 (5)	*0.502 ^#^*
Median serum α-FP at listing in ng/ml (IQR)	12 (5.0–44.0)	17 (6.0–80.7)	9.8 (4.0–28.4)	<0.001 *
Hepatitis status *n* (%) Hepatitis C Hepatitis B Hepatitis B & C No hepatitis	362 (53) 165 (24) 16 (2) 148 (21)	112 (44) 78 (31) 10 (4) 54 (21)	250 (57) 87 (20) 6 (1) 94 (22)	*0.001 ^#^*
*Pathological characteristics at explant*				
Mean tumor number ( ± SD)	2 (1.7)	2 (1.9)	2 (1.5)	0.468 *
Mean size of largest tumor in cm (±SD)	2.7 (1.8)	2.6 (1.6)	2.8 (1.9)	0.659 *
Mean total tumor diameter in cm (±SD)	3.9 (2.9)	3.7 (2.8)	4.1 (3.0)	0.054 *
Capsular invasion *n* (%)	33 (5)	16 (6)	17 (4)	*0.151 ^#^*
Vascular invasion *n* (%) Macrovascular invasion Microvascular invasion No invasion Not available	22 (3) 73 (11) 436 (63) 160 (23)	13 (5) 27 (11) 178 (70) 36 (14)	9 (2) 46 (11) 258 (59) 124 (28)	*0.177 ^#^*
Lymph node status *n* (%) Involved Uninvolved Not available	21 (3) 339 (49) 331 (48)	6 (2) 172 (68) 76 (30)	15 (3) 167 (38) 255 (59)	*0.049 ^#^*

* Mann–Whitney U Test; ^#^ Pearson Chi-Square Test (a *p*-value < 0.05 was considered statistically significant).

**Table 2:** Factors influencing overall survival (Abbreviations: CI – Confidence interval; α-FP—alpha-fetoprotein).

Variables		5y Survival (*n* at Risk)	10y Survival (*n* at Risk)	95% CI	χ^2^	*P* Log-Rank Comparison (Mantel-Cox)
**Age (years)**	<55 (*n* = 312)	81% (244)	72% (136)	15.04–17.18	5.72	0.017
	≥55 (*n* = 379)	75% (272)	67% (121)	13.02–15.15		
**Underlying disease**	Viral hepatitis (*n* = 543)	78% (411)	71% (208)	14.63–16.32	6.08	0.014
	Alcohol-related cirrhosis (*n* = 65)	72% (46)	57% (22)	10.22–10.74		
**Hepatitis status**	Hep B (*n* = 165)	82% (132)	79% (85)	15.57–18.31	7.25	0.007
	Hep C (*n* = 362)	76% (266)	67% (115)	13.31–15.57		
**α-FP**	<10 ng/mL (*n* = 216)	79% (152)	73% (180)	14.99–17.72	4.31	0.038
	≥10 ng/mL (*n* = 228)	75% (64)	63% (94)	12.70–15.11		
**Capsular invasion**	no (*n* = 656)	79% (496)	70% (250)	14.47–16.03	4.21	0.04
	yes (*n* = 33)	61% (20)	48% (6)	8.42–15.46		
**Vascular invasion**	no (*n* = 436)	80% (343)	72% (185)	14.64–16.47	5.712	0.017
	yes microvascular (*n* = 73)	67% (46)	52% (12)	9.93–14.70		
	no (*n* = 436)	80% (343)	72% (185)	14.64–16.47	29.64	<0.001
	yes macrovascular (*n* = 22)	32% (7)	26% (4)	2.92–8.78		

α-FP cut-off of 10 ng/mL based on Ruoslahti and Seppala [16].

**Table 3:** Factors predictive of reduced survival on multivariate Cox Regression stepwise analysis (Abbreviations: HR, hazard ratio; CI, confidence interval; α-FP, alpha fetoprotein).

Step	Variable	HR	95% CI	P
1	age	1.027	1.003–1.052	0.027
2	age	1.029	1.005–1.054	0.018
	α-FP	1.000	1.000–1.000	0.057
3	age	1.027	1.003–1.052	0.027

**Table 4:** Comparison of 5- and 10-year overall survival rates following liver transplantation for hepatocellular carcinoma within the University of California San Francisco (UCSF) criteria from large series around the world.

Author/Year [Ref]	Region	Within UCSF Criteria	
		5-Year Survival	10-Year Survival
Duffy et al. 2007 [26]	USA	64%	n.a.
Lee et al. 2008 [29]	Korea	76%	n.a.
Unek et al. 2011 [28]	Turkey	54%	n.a.
Bonadio et al. 2015 [25]	Belgium	74%	n.a.
Pinero et al. 2018 [27]	Latin America	66%	n.a.
Current study 2022	ANZ	80%	72%

Abbreviations: n.a.—not available; USA—United States of America; ANZ—Australia and New Zealand.

## Data Availability

Authors are unable to provide this data owing to the regulations surrounding the information contained within the ANZ Liver and Intestinal Transplant registry.
